# Early and long-term outcomes of endoscopic submucosal dissection for early gastric cancer in a large patient series

**DOI:** 10.3892/etm.2014.1488

**Published:** 2014-01-17

**Authors:** KEN OHNITA, HAJIME ISOMOTO, SABURO SHIKUWA, HIROYUKI YAJIMA, HITOMI MINAMI, KAYOKO MATSUSHIMA, YUKO AKAZAWA, NAOYUKI YAMAGUCHI, EIICHIRO FUKUDA, HITOSHI NISHIYAMA, FUMINAO TAKESHIMA, KAZUHIKO NAKAO

**Affiliations:** 1 Department of Gastroenterology and Hepatology, Nagasaki University Hospital, Nagasaki-shi, Nagasaki 852-8501, Japan; 2 Sankokai Miyazaki Hospital, Isahaya-shi, Nagasaki 854-0066, Japan

**Keywords:** endoscopic submucosal dissection, early gastric cancer, early outcomes, long-term outcomes

## Abstract

Endoscopic submucosal dissection (ESD) enables the curative resection of early gastric cancer (EGC); however, little information is available on the long-term outcomes of ESD. This study was conducted to clarify the clinical outcomes of a large number of patients with EGC who underwent ESD. The early outcomes were assessed in 1,209 patients and the long-term outcomes were assessed in 300 patients at a follow-up >5 years after the ESD procedure. The overall survival rates were compared between indication and expanded-indication groups, and between the patients who did or did not undergo additional surgery in an out-of-indication group. Overall survival rates were also compared among different age groups. In total, 617 lesions were classed as the indication group, 507 as the expanded-indication group and 208 as the out-of-indication group. Curative resection rates were 96.6% and 91.5% in the indication and expanded-indication groups, respectively. In terms of the long-term outcomes, 20 of the 146 patients in the indication group, 15 of the 105 patients in the expanded-indication group and one of the 23 patients who underwent additional surgery in the out-of-indication group succumbed due to causes other than gastric cancer. Among the 26 patients who did not undergo additional surgery in the out-of-indication group, 10 mortalities occurred, including one due to gastric cancer. The five-year survival rates were not significantly different between the indication and expanded-indication groups. In the out-of-indication group, the five-year survival rate for the patients who did not undergo additional surgery (65.0%) was significantly lower than that for those who did undergo additional surgery (100%) (P<0.01). The five-year survival rate of patients aged >80 years (67.1%) was significantly lower than that of the younger patients (<60 years, 91.6%; sixties, 93.0%; seventies, 84.5%) (P<0.0001). In conclusion, although expanded-indication of ESD for EGC is appropriate, comorbidities require consideration in elderly patients.

## Introduction

Early gastric cancer (EGC) is defined as gastric cancer that is confined to the mucosa or submucosa (T1 cancer), irrespective of the presence of regional lymph node metastases (1). In 1995, it was reported that almost 10,000 cases of EGC are detected annually in Japan, accounting for 40–50% of all gastric cancers (2).

Endoscopic submucosal dissection (ESD) is widely recognized as a safe and effective treatment for EGC (3–6). However, little information is available concerning the long-term outcomes of ESD in large numbers of patients.

The gold standard study design for evaluation of the efficacy of endoscopic treatment of EGC is a long-term, large-scale, randomized controlled trial. The excellent prognosis following surgical treatment of EGC, particularly in cases indicated for endoscopic resection, makes randomized controlled trials unethical. Therefore, the feasible evidence of the efficacy of EMR/ESD comes from long-term clinical follow-up data.

In the present study, the clinical outcomes of a large number of patients with EGC who underwent ESD were investigated.

## Patients and methods

### Patients

A total of 1,332 EGCs in 1,209 consecutive patients were treated by ESD at the affiliated hospitals of Nagasaki University Hospital from January 2001 to December 2010. The indications for ESD were determined by the presence or absence of nodal metastasis (7–9) and according to the criteria for endoscopic resection proposed in the Treatment Guidelines for Gastric Cancer in Japan (10). The indication criteria were defined as differentiated-type mucosal cancer without ulceration, ≤20 mm in diameter. The expanded-indication criteria were defined as follows: Differentiated-type mucosal cancer without ulceration, irrespective of tumor size; differentiated-type mucosal cancer with ulceration, ≤30 mm in diameter; differentiated-type minute (<500 μm from the muscularis mucosae) submucosal invasive cancer, ≤30 mm in diameter; and undifferentiated-type mucosal cancer without ulceration, ≤20 mm in diameter with no lymphovascular involvement. The out-of-indication criteria were defined as EGCs that did not meet the indication criteria or the expanded-indication criteria. Written informed consent was obtained from each patient.

### ESD

The EGCs were first identified and demarcated using white-light endoscopy and chromoendoscopy with indigo carmine solution, after which marking around the lesions was performed by cautery with a needle knife. Glycerol (10% glycerol and 5% fructose; Chugai Pharmaceutical Co. Ltd., Tokyo, Japan) or MucoUp^®^ (Johnson and Johnson Co. Ltd., Tokyo, Japan) were then injected into the submucosal layer to lift the mucosa. A circumferential mucosal incision was made around the lesion using an insulation-tipped (IT) Knife 2 (Olympus Medical Systems Corp., Tokyo, Japan) or a Flush Knife (Fujifilm Corp., Tokyo, Japan). Submucosal dissection was performed using the IT Knife 2, a Hook Knife (Olympus Medical Systems Corp.) or the Flush Knife to achieve complete removal of the lesion. High-frequency generators (ICC 200 or VIO 300D; ERBE Elektromedizin GmbH, Tübingen, Germany) were used during marking, incision of the gastric mucosa and exfoliation of the gastric submucosa.

### Early outcomes

Early outcomes (perforation, bleeding rate and curability) were assessed for the 1,332 EGCs in the 1,209 consecutive patients. The patients included 882 men and 327 women, with a mean age of 72 years (range, 33–95 years). Perforation was diagnosed endoscopically or by the presence of free air on an abdominal plain radiograph or computed tomography scan. Procedure-associated bleeding was defined as bleeding that required transfusion or surgical intervention, or bleeding that caused the hemoglobin level to decrease by 2 g/dl (11).

*En bloc* resection refers to a resection in one piece (11). The curability of ESD was classified as either curative or non-curative (11,12). Resections were deemed curative when a tumor was excised *en bloc* and was within the indication or expanded-indication criteria with tumor-free lateral and vertical margins and no lymphovascular invasion. When histological evaluation was challenging or identified that a lesion was outside the indication or expanded-indication criteria and/or that it had a positive margin or lymphovascular invasion, the curability was defined as non-curative.

### Long-term outcomes

Long-term outcomes were assessed for a total of 342 EGCs in 319 consecutive patients treated using ESD from January 2001 to December 2005. The follow-up was conducted >5 years after the procedure. The patients included 224 men and 95 women, with a mean age of 71 years (range, 33–92 years). Nineteen patients had multiple EGCs with a total of 42 lesions. In the analysis of long-term outcomes, 300 patients were enrolled due to the exclusion of the 19 patients with multiple EGCs. The overall survival rates were compared between the indication and expanded-indication groups, and between the patients who did or did not undergo additional surgery in the out-of-indication group. The overall survival rates among different age groups were also compared.

### Statistical analysis

The statistical significance of the differences with respect to each complication was determined using Fisher’s exact test or the χ^2^ test. Data for the long-term outcomes were calculated using the Kaplan-Meier method and analyzed by the log-rank test. P<0.05 was considered to indicate a statistically significant difference. This study was approved by the ethics committee of Nagasaki University Hospital.

## Results

### Early outcomes

Table I categorizes the resected lesions by histopathological examination. The indication criteria included 617 (46.3%) lesions; 507 (38.1%) lesions were included in the expanded-indication criteria and 208 (15.6%) lesions were included in the out-of-indication criteria. Table II lists the early outcomes of the patients. The curative resection rates for *en bloc* resection were 96.6% (596/617) and 91.5% (464/507) in the indication and expanded-indication groups, respectively. The total perforation rate was 2.9% (39/1,332), and the rate for the expanded-indication group was significantly higher than that for the indication group [4.3% (22/507) versus 1.8% (11/617), P<0.05; Table III]. Only one case of late-onset perforation required surgery. Overall, the ESD-associated bleeding rate was 2.0% (26/1,332) and it was significantly higher for the out-of-indication group [4.8% (10/208)] compared with the indication group [1.1% (7/617)] (P<0.01) or the expanded-indication group [1.8% (9/507)] (P<0.05; Table IV). The complication rate was similar to those calculated in other studies (13–15). Local recurrence was identified in two patients and the lesions in these patients were excised with piecemeal resection.

### Long-term outcomes

**Figure 1:**
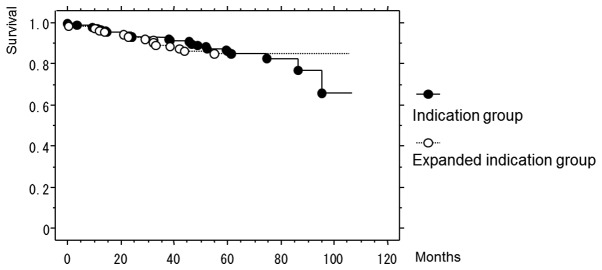
Five-year survival rates of the indication and expanded-indication groups.

**Figure 2:**
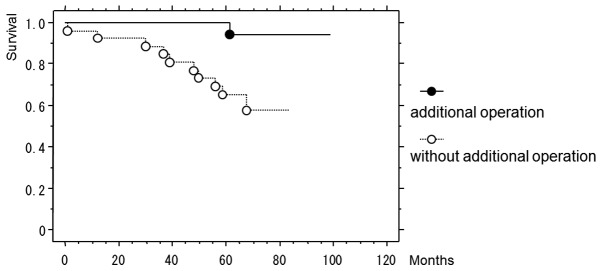
Five-year survival rates of the patients who did or did not undergo additional surgery in the out-of-indication group.

**Figure 3:**
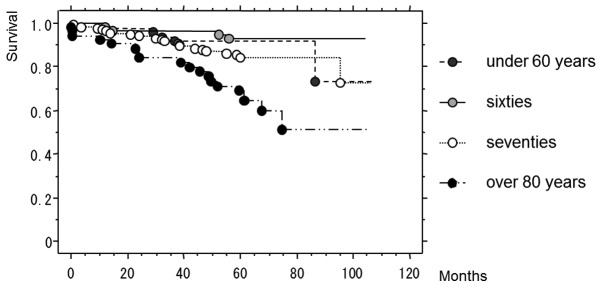
Five-year survival rates of the younger age groups (<60 years old, sixties and seventies) and the older age group (>80 years old).

Patients (n=319), consisting of 224 men and 95 women, with a mean age of 71 years (range, 33–92 years), were followed up >5 years after ESD was performed. Of those, 19 patients (42 lesions) had multiple EGCs; thus, in the analysis of long-term outcomes, the other 300 patients were enrolled. The median follow-up term of observation was 66 months, ranging from 1 to 106 months. Table V presents the categorization of the resected lesions that were enrolled in the long-term outcome analysis. Of the 49 patients in the out-of-indication group, 23 underwent additional surgery and 26 did not due to advanced age, concomitant diseases and/or rejection of surgery by the patient. Of the 146 patients in the indication group, 20 passed away; 18 mortalities were due to diseases other than gastric cancer and two were due to unknown causes. Of the 105 patients in the expanded-indication group, 15 patients succumbed to diseases other than gastric cancer. Of the 23 patients who underwent additional surgery in the out-of-indication group, one mortality occurred, which was not due to gastric cancer. Among the 26 patients who did not undergo additional surgery in the out-of-indication group, 10 mortalities occurred, including one due to gastric cancer. The five-year survival rate was not significantly different between the indication and expanded-indication groups (Fig. 1). However, in the out-of-indication group, the five-year survival rate of the patients who did not undergo additional surgery (65.0%) was significantly lower than that of the patients who did undergo additional surgery (100%) (P=0.0062; Fig. 2). In the analysis by age, the five-year survival rate of the patients >80 years old (67.1%) was significantly lower than those of the younger age groups (<60 years old, 91.6%; sixties, 93.0%; seventies, 84.5%) (P<0.0001; Fig. 3).

## Discussion

Gotoda *et al* (7) suggested that early gastric cancer with no risk of lymph node metastasis is definable by using a large database, so the indications for endoscopic treatment have been expanded in Japan. *En bloc* resection of ESD provides a much higher curative resection rate than piecemeal resection via endoscopic mucosal resection (EMR) (11). In addition, ESD permits precise histopathological examination for the assessment of curability to guide further management and to stratify the risk of a patient developing metastases. Successful outcomes are therefore enabled following ESD (6,16). In the present study, two cases of EGC local recurrence were excised via piecemeal resection of ESD. It is important to be able to excise via *en bloc* resection. ESD is an excellent procedure as it decreases the incidence of local recurrence following *en bloc* resection more effectively than EMR (17). The development of ESD has allowed the indications for endoscopic treatment to be extended. In the present study, although the perforation rate was higher for the expanded-indication group than for the indication group, most of the perforations were controlled endoscopically.

Although endoscopic treatment is an accepted therapy for EGC in Japan (3–6), there have been few studies concerning the long-term outcomes (18,19). The present study has demonstrated that the survival rate of patients meeting the expanded-indication criteria has been similar to that of patients meeting the indication criteria. Therefore, the current expanded indications may be appropriate. In the present study, mortalities due to gastric cancer were not observed in the patients in the indication and the expanded-indication groups. However, 13.9% (35/251) of the patients in these two groups succumbed to diseases other than gastric cancer. In particular, the survival rate of the patients >80 years old was poor; most of the patients >80 years of age succumbed to non-cancerous diseases such as pulmonary disease, heart disease and cerebral infarction. As may be expected, the life expectancies of the older patients were shorter than the life expectancies of the younger patients. This result may be a natural outcome; however, there are a number of studies that suggest ESD is effective even in elderly patients (13,18,19). The natural history of EGC has been poorly delineated. Therefore, the degree to which EGC improves the prognosis of elderly patients, regardless of the presence or absence of a therapeutic intervention, has not been fully clarified. In the present study, a number of the elderly patients had concomitant diseases, but the mortalities of a number of the others were unpredictable. Although it is difficult to decide whether elderly patients with EGC should undergo ESD, this issue merits careful thought. When the lesions are judged as out-of-indication according to histopathological analysis, additional surgery with lymph node dissection is recommended. In the present study, in the out-of-indication group, the five-year survival rate of the patients who did not undergo additional surgery was significantly lower than that of the patients who underwent additional surgery. The reasons for not having additional surgery included advanced age and comorbid disease, and such patients may not require ESD. However, as a number of elderly patients may live for a long time, it is difficult to decide whether ESD should be performed. This is an issue for future study.

In conclusion, the results of the present study demonstrated that the early and long-term outcomes of ESD for patients meeting the expanded-indication criteria are similar to those of patients meeting the indication criteria. When deciding whether to perform ESD in elderly patients, it is necessary to consider the presence of underlying comorbid diseases.

## Figures and Tables

**Table I:** All EGC cases categorized by pathology (n=1,332).

Criteria	Lesions [n (%)]
Indication [differentiated M UL(−) ≤20 mm]	617 (46.3%)
Expanded indication	507 (38.1%)
Differentiated M UL(−) >20 mm	293
Differentiated M UL(+) ≤30 mm	146
Differentiated SM1 ≤30 mm	62
Undifferentiated M UL(−) ≤20 mm	6
Out of indication	208 (15.6%)

M, mucosal cancer; UL, ulceration; (−), without; (+), with; SM1, submucosal invasive cancer.

**Table II:** Early outcomes.

Outcomes	Indication (n=617)	Expanded indication (n=507)	Out of indication (n=208)
*En bloc*	605/617	487/507	199/208
	98.1%	96.1%	95.7%
Curative	596/617	464/507	-
	96.6%	91.5%	
Piecemeal or non-curative	21/617	43/507	208/208
	3.4%	8.5%	100%

**Table III:** Perforation rates.

Criteria	Perforation rate [% (n/total)]
Indication	1.8% (11/617)
Expanded indication	4.3% (22/507)
Out of indication	2.9% (6/208)
Total	2.9% (39/1332)

The perforation rate was significantly higher in the expanded-indication group than in the indication group (P<0.05).

**Table IV:** ESD-associated later bleeding rates.

Criteria	Bleeding rate [% (n/total)]
Indication	1.1% (7/617)
Expanded indication	1.8% (9/507)
Out of indication	4.8% (10/208)
Total	2.0% (26/1332)

The ESD-associated later bleeding rate was significantly higher in the out-of-indication group compared with the indication group (P<0.01) or the expanded-indication group (P<0.05).

**Table V:** Cases followed up >5 years after ESD, with the exception of those with multiple lesions (n=300).

Criteria	Patients (n)
Indication (differentiated M UL(−) ≤20 mm)	146
Expanded indication	105
Differentiated M UL(−) >20 mm	72
Differentiated M UL(+) ≤30 mm	21
Differentiated SM1 ≤30 mm	10
Undifferentiated M ≤20 mm	2
Out of indication:	49
Additional surgery (+)	23
Additional surgery (−)	26

M, mucosal cancer; UL, ulceration; (−), without; (+), with; SM1, submucosal invasive cancer.
